# Assessment of advanced glazing systems for building energy efficiency in hot-arid climates

**DOI:** 10.1038/s41598-026-46722-4

**Published:** 2026-04-13

**Authors:** Ahmad I. Elshamy, Yousef Elgefly, Yara El-Metwally, Serag Salem

**Affiliations:** 1 Mechanical Engineering Department, The British University in Egypt, El Sherouk, Egypt; 2 Architectural Engineering Department, The British University in Egypt, El Sherouk, Egypt

**Keywords:** Building thermal efficiency, Glazing systems, Cooling energy consumption, Simulation model, Energy science and technology, Engineering, Materials science

## Abstract

The built environment is considered among the most contributing factors to energy consumption. The building envelope plays a crucial role in determining the building energy consumption, regulating heat transfer and maintaining adequate indoor environmental quality. Hence, optimizing the thermal performance of the building envelope by achieving optimal glazing solutions while fulfilling the Sustainable Development Goal (SDG 7) is the main aim of this research. This study investigates the impact of various facade glazing systems on the building energy performance, focusing on the cooling energy consumption of an office building in a hot arid climate, Cairo, Egypt. The novelty of this study lies in evaluating the adaptability of multiple glazing technologies in hot arid climate employing two different approaches: experimental and simulation. A thorough literature review was first conducted to have a comprehensive background, followed by an experimental study. The experimental setup involved small concrete chambers equipped with temperature sensors that were utilized to test four different glazing types: single-glazed clear glass, double-glazed clear glass, low-emissivity coated Vistalite sky-blue double-glazed pane, and solar control Stopray Smart 30 double-glazed pane. A simulation model was then established using TRNSYS 17.0.0 software to validate the experimental findings. A deviation of less than 9% was detected. Subsequently, a comprehensive simulation case study on a medium office building in Cairo was performed. The experimental results demonstrated that the solar control glazing (Stopray Smart 30) exhibited the best performance, reducing the internal chamber temperature by 17 °C compared to ordinary clear glass and enhancing thermal efficiency by 29%. The simulation results indicate a significant enhancement of the building thermal efficiency by 22% and a remarkable reduction of cooling energy consumption by 50% when replacing conventional clear double-glazed windows with solar control glazing (Stopray Smart 30). This research provides crucial insights into enhancing energy efficiency in buildings in hot climates through the optimal selection of high-performance glazing systems.

## Introduction

The exponential increase in energy demand, global warming and climate change are the primary concerns of our world nowadays. The building sector is the highest contributor to these environmental threats; it demands an enormous amount of energy during its life cycle. Commercial and residential buildings employ about 40–50% of their total energy consumption in heating, air conditioning (HVAC) and ventilation according to International Renewable Energy Agency (IRENA)^1^. Studies show that residential, commercial and governmental sectors in Cairo consume over 60% of the total electricity, which contributes the highest share among the other sectors. The air conditioning for residential and commercial sectors in Egypt consumes about 15.6% of the total electricity consumption^2^.

The building envelope is the boundary between the indoor and outdoor environment and is the main source of heat transfer. It significantly impacts the building’s energy performance and contributes substantially to HVAC loads to maintain an adaptable indoor environmental quality. Most research consider that an efficient building envelope has a significant impact on obtaining thermal comfort and can reduce the energy consumption by 40–50%^3^.

Therefore, finding innovative solutions to enhance building energy performance and minimize cooling and heating loads across all industries is a critical target. Adaptive envelopes may integrate efficient insulation materials, smart glazing systems, and advanced technologies to optimize the occupants’ thermal comfort and minimize energy consumption.

## Literature review

Although transparent building facades provide us with daylight and ventilation, they are the main source of heat transfer. Therefore, regulating indoor air temperature without compromising occupant comfort is necessary. Previous studies show that building envelopes using conventional glazing systems account for about 20–25% of heat transmittance in buildings^4^. The higher the window-to-wall ratio, the higher the heat radiation transferred through it^5^. Studies show that fenestration with low performance possesses a thermal transmittance of U-value that ranges from 2 W/m2K up to 4.5 W/m2K. This range can be minimized to a range from 0.5 to 1.5 W/m2K by replacing ordinary windows with smart glazing systems, which is still much larger than the U-value of walls and roofs^6,7^ Hence, optimizing heat gain in summer and loss in winter in the context of the climatic zone may reduce building energy consumption for heating and cooling^8^.

Integrating innovative glazing technologies is one of the most important strategies that can elevate building thermal performance and reduce energy demands in buildings. Bhattacharjee et al. investigated the impact of replacing the balcony single glazing with double-pane glazing on the building energy consumption in cold climates. The results show a decrease of 10% in the energy consumption due to the increase in thermal insulation by using Double-pane glazing. It reduces solar energy transmittance and consequently, carbon emissions and heating costs^9^.

Several studies introduced various glazing techniques to enhance the heat transmittance of glazing units, such as using multilayer glazing structure: single, double, triple, quadruple and hexagonal glass^10,11^. Adding Low-emissivity coated substances and tints on the surface of the glass^12,13^, and adding participating mediums in the glazing cavity: air^14^, CO2, HFC-125^15^, water^16^, aerogel^17^, silica aerogel^18^, phase change material^19^ are commonly used techniques to improve efficiency of glazing units. A recent study by Chen et.al demonstrates the significant impact of triple-glazing on indoor thermal comfort by reducing heat transfer significantly. Triple-glazing systems are a key component of sustainable building practices, which promote environmentally responsible buildings^20^.

**Fig. 1:**
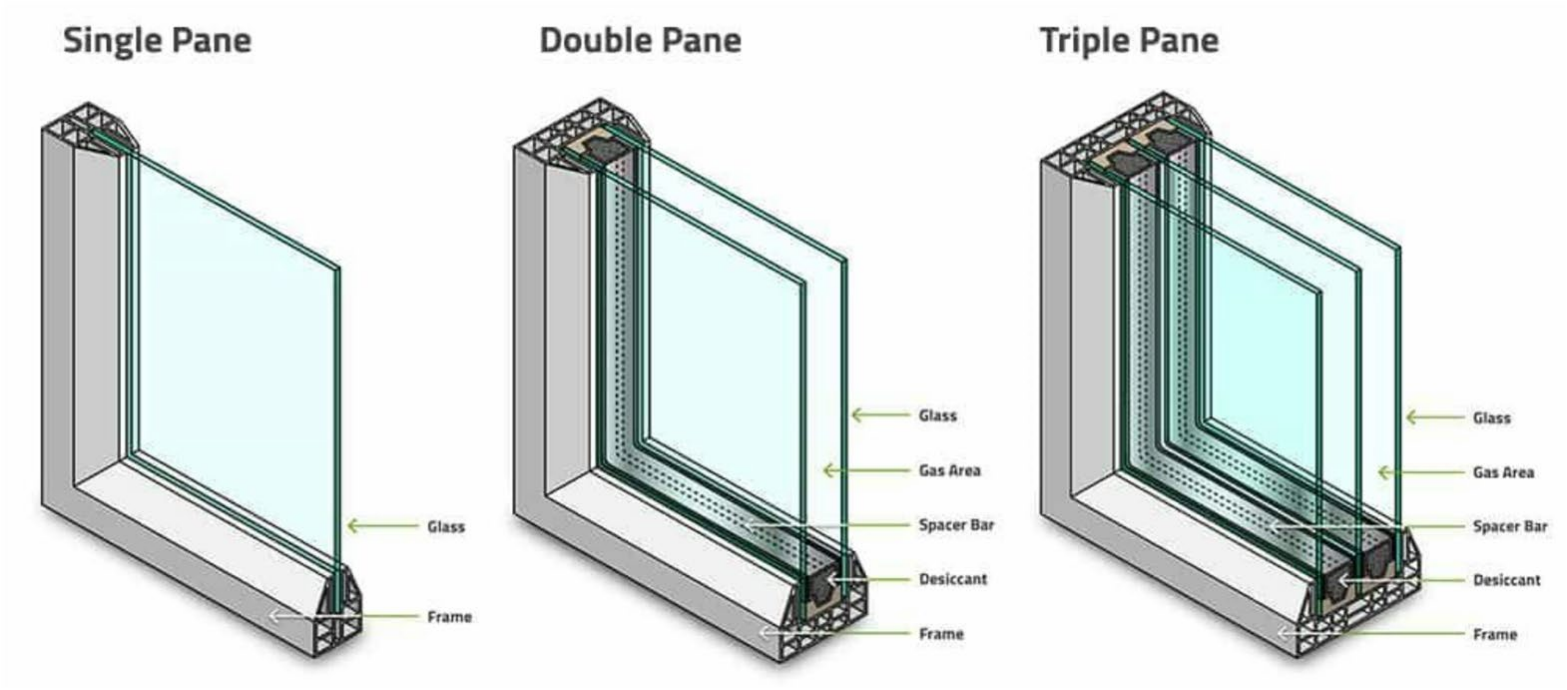
Different glazing systems^21^.

According to a recent study by Müslüm et al., changing windows from a double pane to a triple pane window, as shown in Fig. 1, reduced the energy consumption by 50 to 67%^21^. El-Darwish and Gomaa^22^ studied the relationship between multiple retrofitting variables and the energy consumption in three Egyptian universities in Alexandria, Gharbeia and Beheira. Their results demonstrated that using double glazing with Argon filling saved energy by 8%.

Abdin et al.^23^ created a simulation model to study the impact of using different glass types on the energy consumption of an office building in 6th of October City, Egypt. Abdin et al. concluded that using glass treated with nanotechnology as an energy efficiency solution to reduce solar gain through glazing can achieve 20% reduction in energy consumption when compared to the base case with 6 mm thickness clear glass.

Currently, most studies and experiments focus on building energy saving in cold climates. However, this research aims to enhance energy efficiency of glazed buildings in hot arid climates, specifically in Cairo, Egypt. A model of a medium office building was created to evaluate the impact of various window glazing types on the energy consumption for cooling.

## Experimental work

**Fig. 2:**
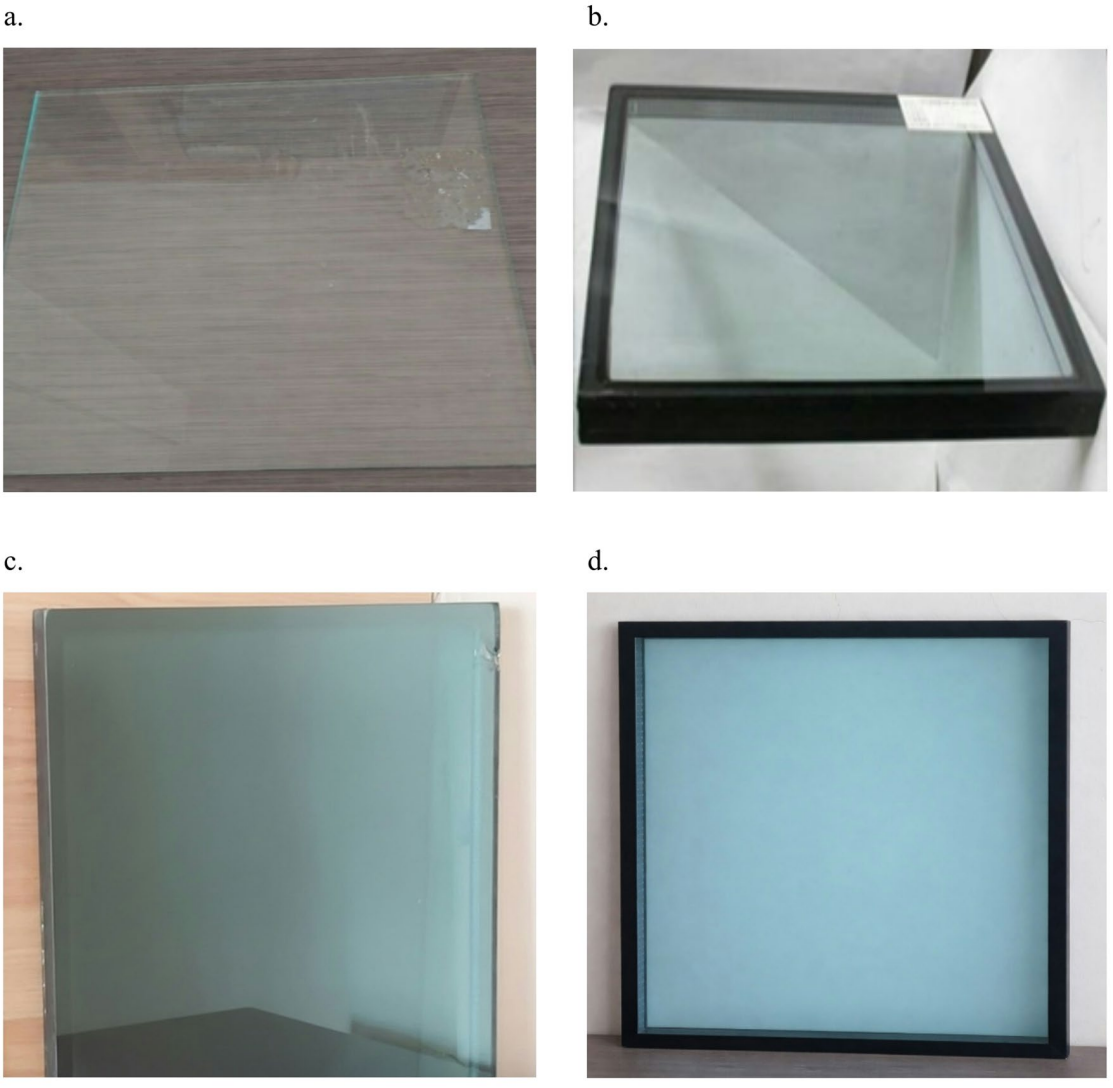
Proposed glazing systems (**a**) normal clear glass, (**b**) double glazed clear glass, (**c**) Vistalite sky-blue, and d. Stopray Smart 30, [author,2025].

The applied study investigates the energy performance of an existing office building with various glazing systems. Four different types of glazing were examined: Single glazed clear glass, Double glazed clear glass, Stopray Smart 30 coated double-glazed pane, and Vistalite sky blue double-glazed pane. Stopray Smart 30 consists of two glass panes with thickness of 6 mm and a spacing of 12mm, where one is coated with solar control coating and the other is clear float glass. On the other hand, Vistalite sky blue double-glazed pane consists of glass panes 6 mm thickness each separated by 12 mm gap where one pane is low-E coated and the other is clear float glass pane. Figure 2 shows the different applied glazing systems. Table 1 illustrates the optical properties for the introduced systems.

**Table 1:** Optical properties for proposed glazing units.

Name	No. of panes	Solar heat gain coefficient	Solar factor	U value W/m^2^K	Visible light transmittance
clear glass	Single pane	0.86	–	5.9	–
clear glass	Double pane	0.85	–	2.7	–
Vistalite sky blue	Double pane	0.5	0.51	5.7	0.44
Stopray Smart 30	Double pane	0.18	0.17	1.17	0.24

**Fig. 3:**
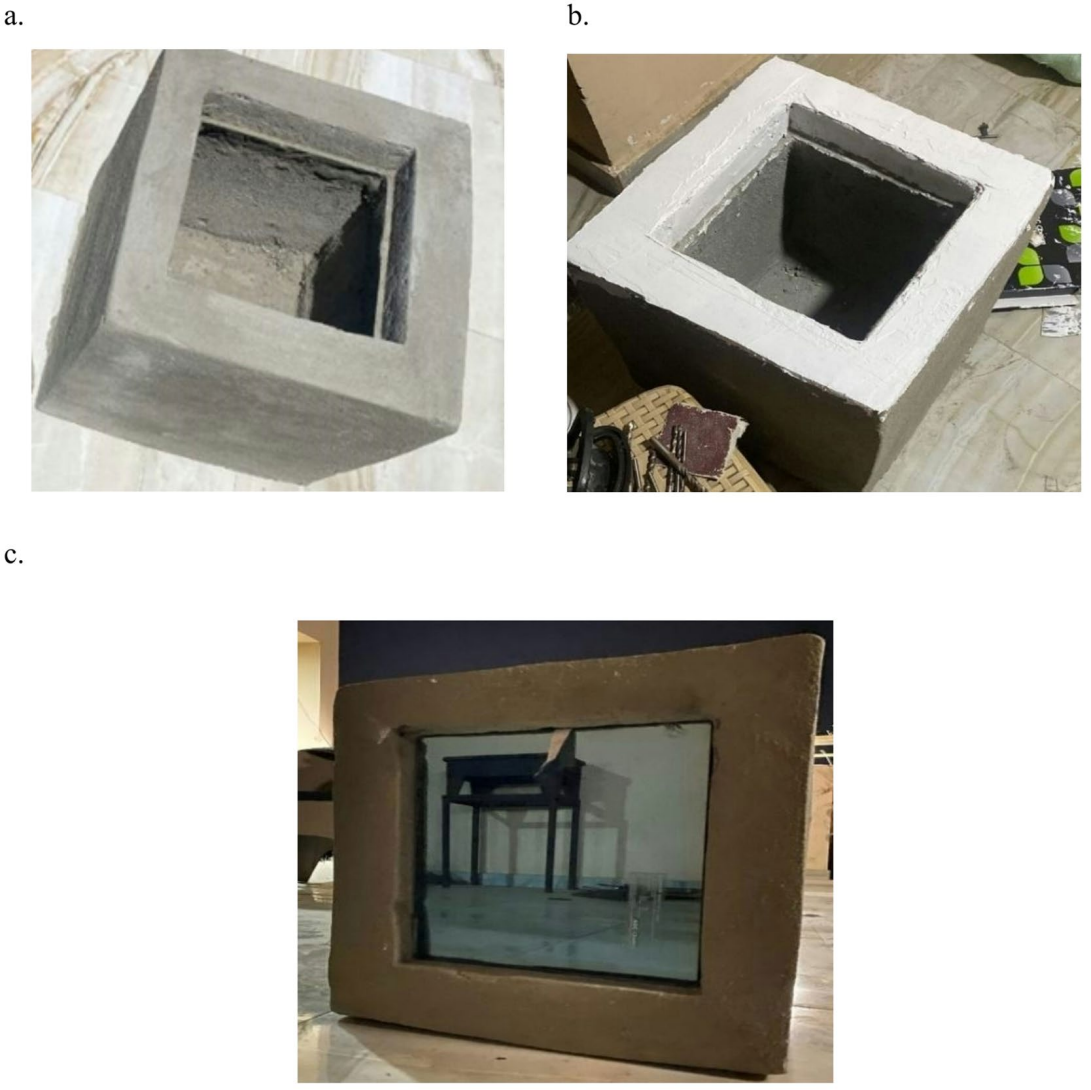
Chamber made from cement and bricks a. before grinding, b. after grinding, and c. after installing the glass system [the author, 2025].

The proposed systems are installed on small chambers manufactured from bricks and cement which are the traditional local building materials in Egypt, as shown in Fig. 3. The chambers with the dimensions of 30*40*30 cm were sanded using sandpaper in order to ensure that the glass is properly fixed to the chamber, Fig. 3b. Finally, the glass system was fixed to the concrete chamber using wall paste to ensure proper sealing of the chamber, see Fig. 3c.

**Fig. 4:**
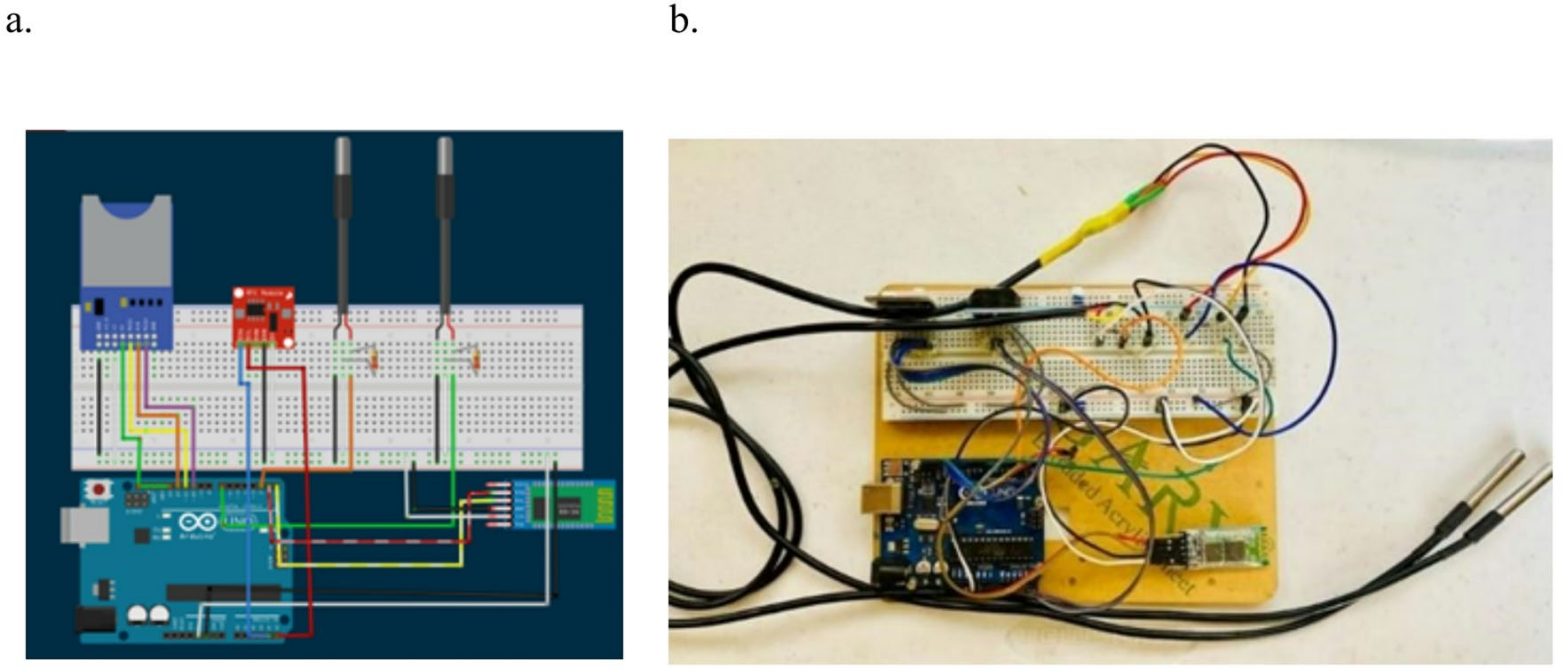
Temperature logger system, (**a**) schematic drawing and (**b**) fabricated unit [author, 2025].

**Fig. 5:**
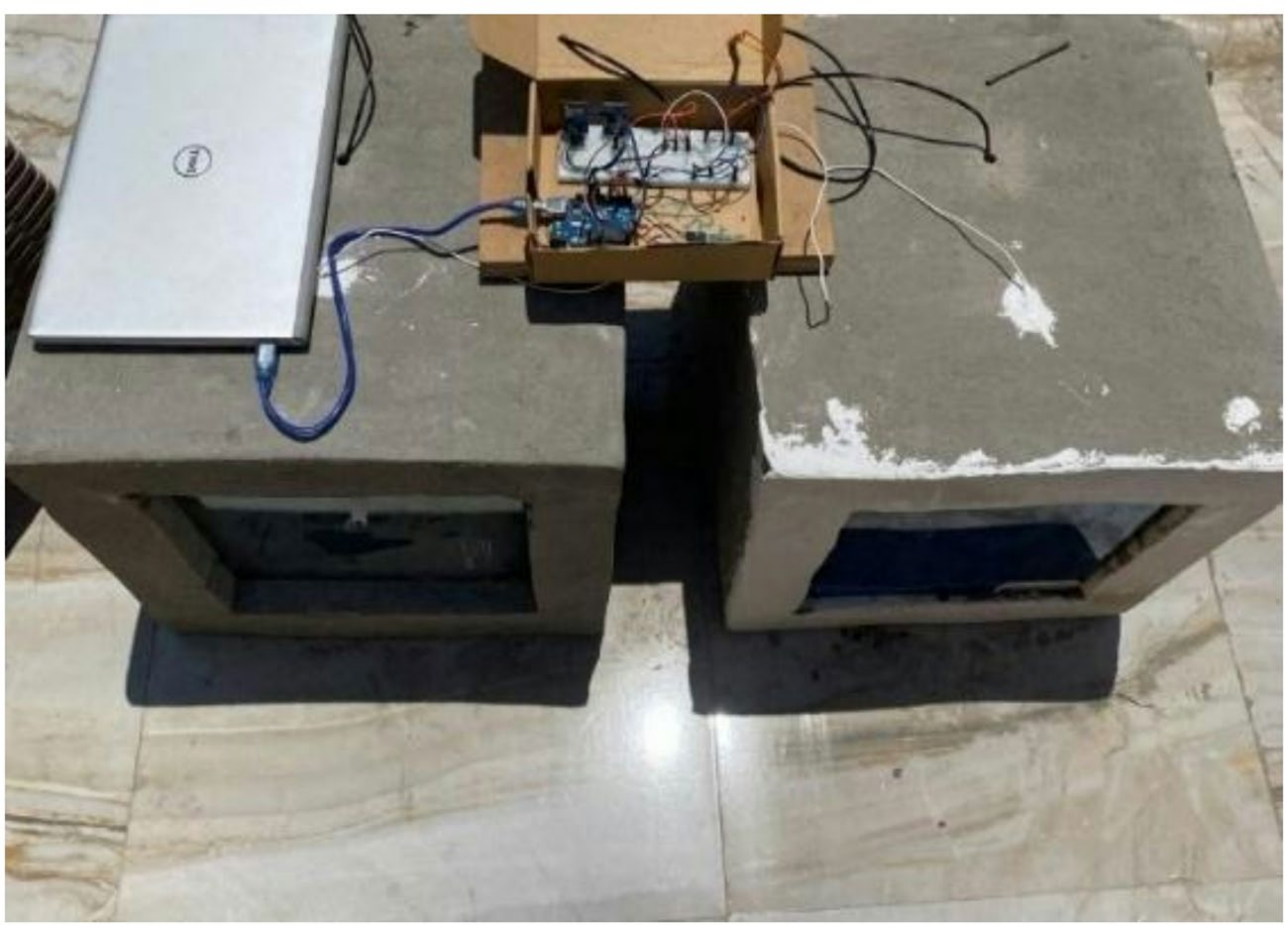
Full experimental setup to test the performance of the glazing units [author, 2025].

Temperature sensors were then installed in the chambers. These sensors were connected to a temperature logger system used for monitoring and saving the temperature readings obtained from the box, Fig. 4. An Arduino temperature logger system is used to monitor and record the temperature variation along the day while remaining in the same location of the building, as illustrated in Fig. 5. Temperature readings act as an indication of the glazing performance.

## Simulation

In the second part of the experimental work, TRNSYS 17.0 software was employed to analyze the energy performance of an office building. It is a simulation programme primarily used in building simulation. The established simulation aims to validate the experimental results obtained from the previous stage by comparing it with the simulation under the same conditions and orientation.

TRNSYS was applied for two main purposes; first, to validate the experimental work by mimicking the chamber used in the experimental analysis. After validation, the proposed glazing system is applied for an existing office building as a case study in order to investigate its impact on the building’s thermal performance.

### Simulation model validation

To ensure the reliability of the numerical results, the TRNSYS 17 simulation model was validated against experimental field measurements. The validation process involved comparing the predicted hourly indoor air temperatures with those recorded during the experimental study over a typical summer day in July. The accuracy of the model was quantitatively assessed using two primary statistical indicators: the Root Mean Square Error (RMSE) Eq. (1) and the Mean Absolute Error (MAE) Eq. (2). These metrics provide a robust measure of the deviation between the simulated (Tsim) and experimental (Texp), ensuring that the numerical framework effectively captures the thermal dynamics of the building envelope under real-world boundary conditions.

1 $${\mathrm{RMSE}} = \sqrt {\frac{1}{n}\mathop \sum \limits_{i = 1}^{n} \left( {T{\mathrm{sim}},i - T{\mathrm{exp}},i} \right)2}$$

2 $${\mathrm{MAE}} = \frac{1}{n}\mathop \sum \limits_{i = 1}^{n} { mid }T{\mathrm{sim}},i - T{\mathrm{exp}},i{ mid }$$

**Fig. 6:**
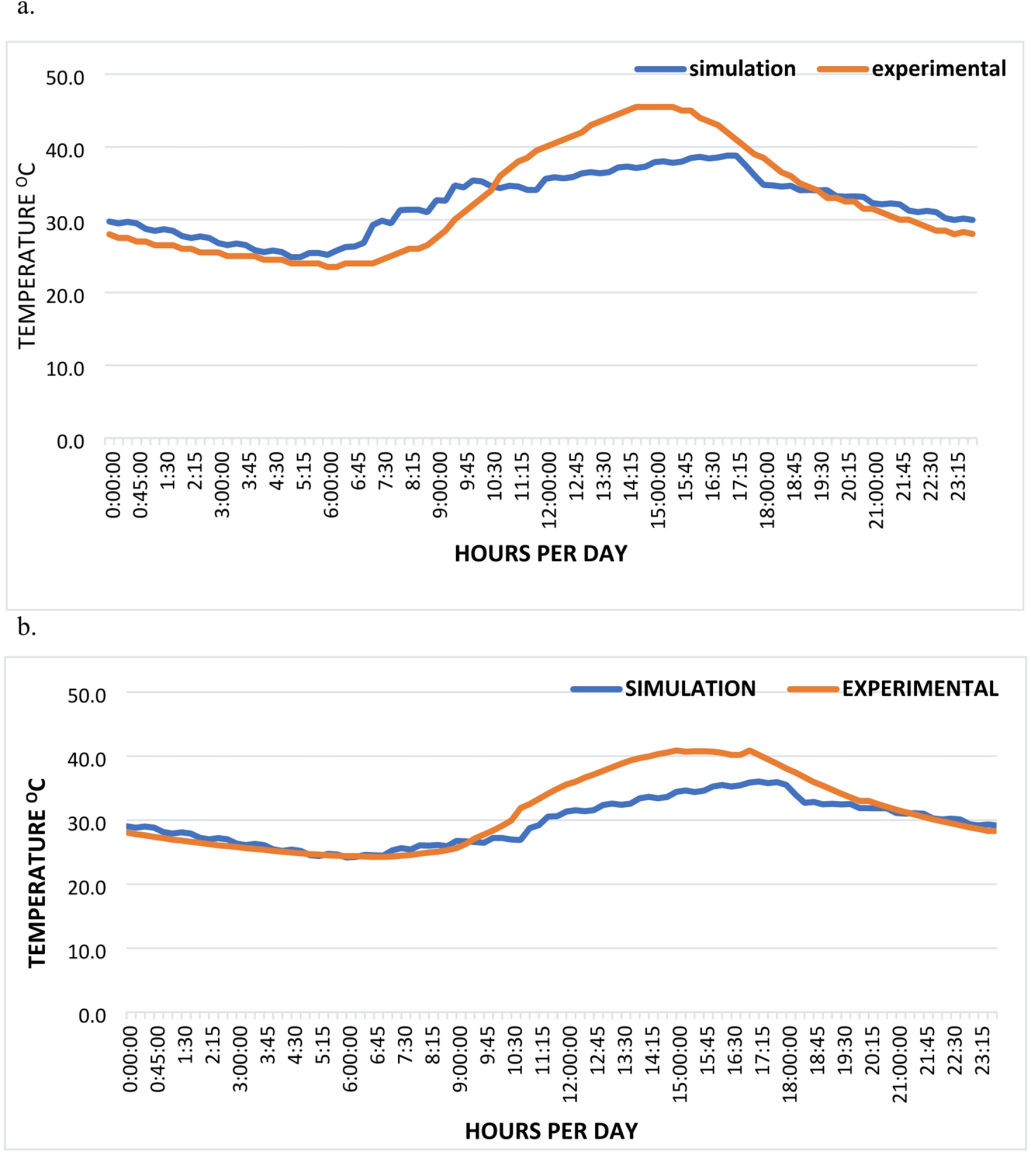
Graph comparing the results obtained from the experiment and the simulation (**a**) low E coating glazing (Vistalite sky-blue), and (**b**) solar control glazing (Stopray Smart 30) [author, 2025].

For the room equipped with Vistalite sky-blue glazing (low-E coating), the simulation model demonstrated a strong correlation with the experimental data as shown in Fig. 6a, yielding an RMSE of 3.75 °C and an MAE of 3.03 °C. The model exhibited high precision during the cooling and night-time phases (20:00 to 06:00), with deviations typically remaining within 1.5 °C. Although the simulation slightly underestimated the peak temperature, predicting a maximum of 39.0 °C compared to the experimental peak of 45.5 °C, the overall thermal trend and the timing of temperature fluctuations were successfully replicated. This level of agreement confirms the model’s capability to simulate the thermal behavior of low-E glazing systems in hot and arid climates.

Similarly, the validation results for the Stopray Smart 30 solar control glazing showed a high degree of consistency between numerical and physical studies as shown in Fig. 6b. The statistical analysis revealed an RMSE of 3.76 °C and a slightly improved MAE of 2.80 °C. The simulation was particularly accurate during the early morning and late evening hours, where the predicted and measured curves nearly overlapped. While a maximum deviation of approximately 7.0 °C was observed during the period of peak solar gain at 15:45, the model accurately captured the lower temperature baseline characteristic of high-performance solar control glazing. These results indicate that the developed simulation model is a reliable tool for predicting the energy-saving potential of advanced glazing technologies in this research context.

### Case study building

After validation, a simulation case study on an existing medium seven-story office building was conducted to investigate its energy performance with the different glazing systems over a whole year. The building is located in Cairo, Egypt.

Two different glazing units are introduced in this part; solar control glazing units (Stopray Smart 30) and double-glazed clear glass unit.

**Fig. 7:**
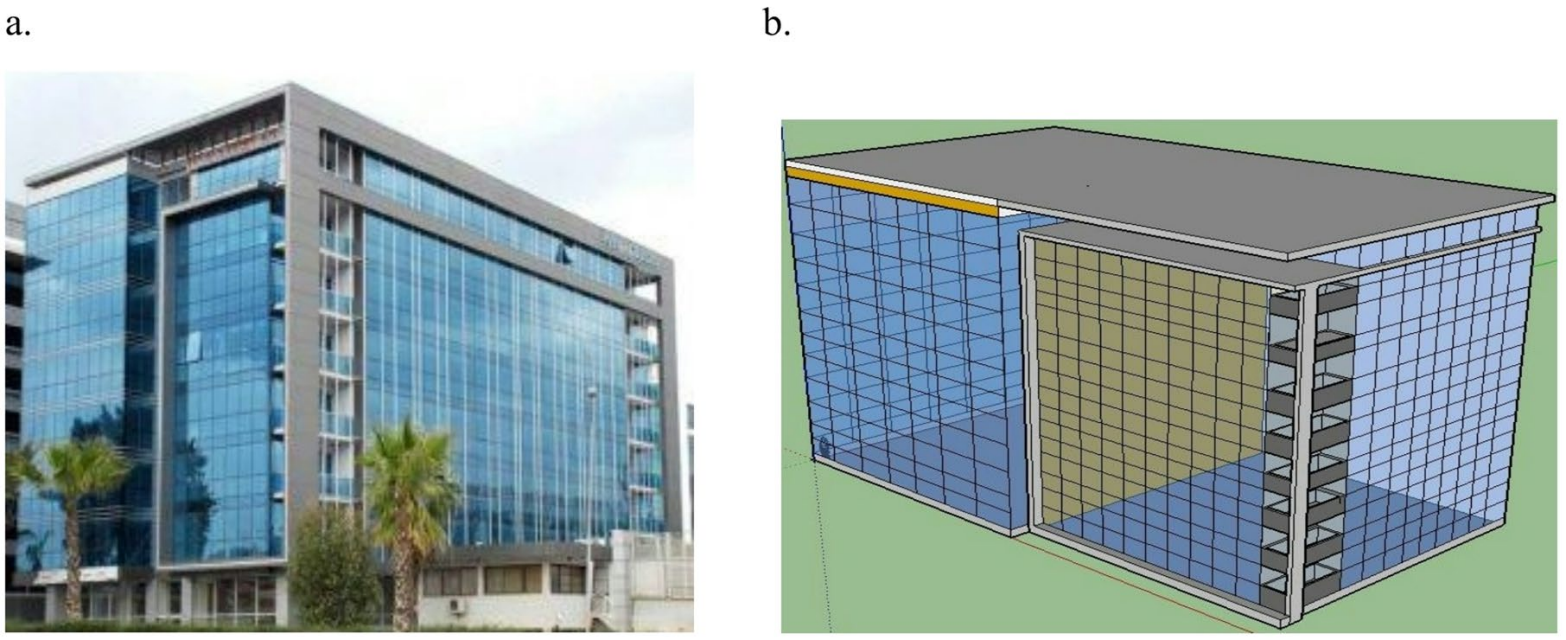
(**a**) Proposed administrative building, (**b**) building sketch [author, 2025].

The proposed building was modeled using SketchUp pro14, as illustrated in Fig. 7.

## Results and discussion

### One-day thermal performance and glazing comparison

**Fig. 8:**
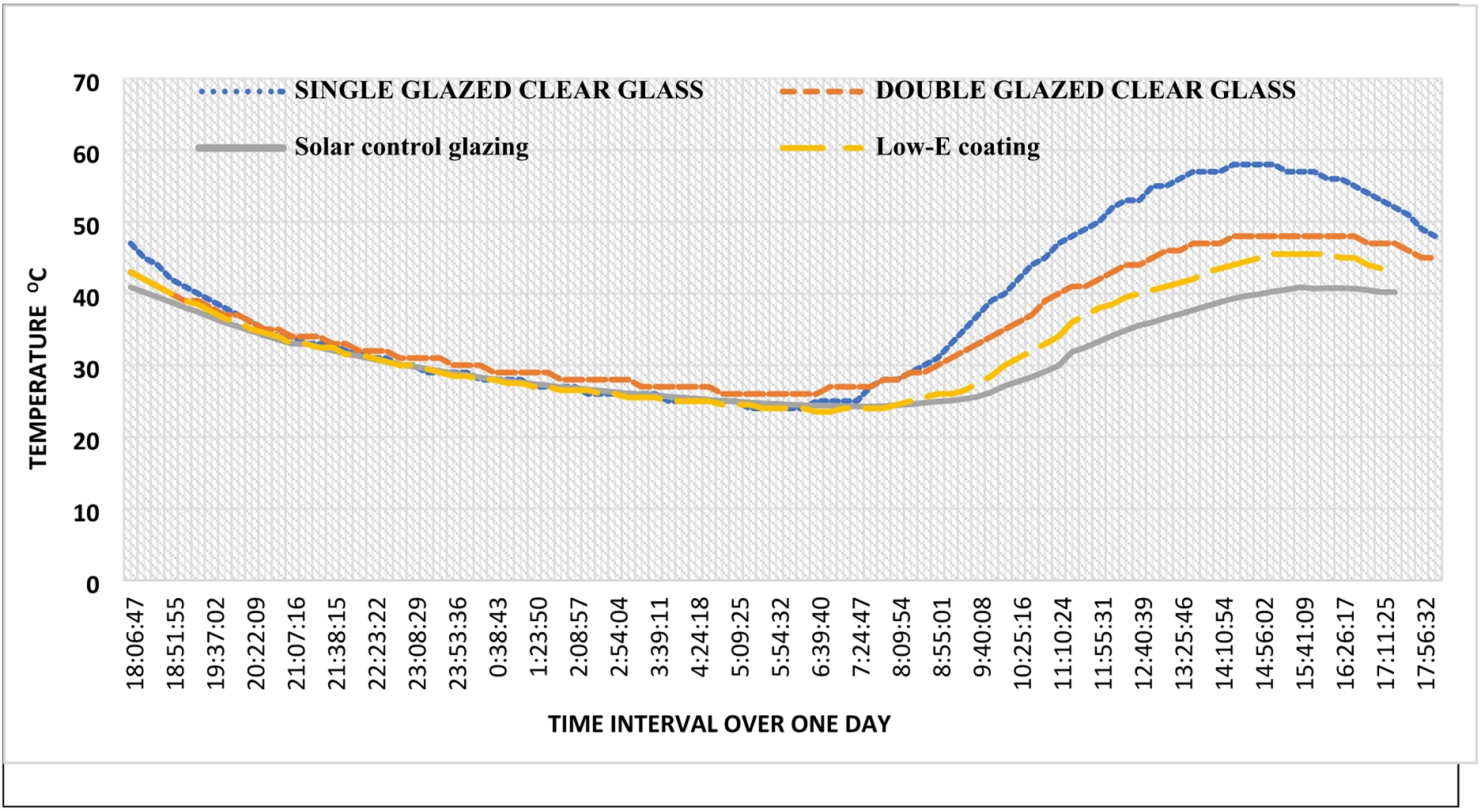
Window efficiency for single clear glass, double clear glass, low E coating glazing (Vistalite sky-blue), and d. solar control glazing (Stopray Smart 30).

The comparative impact of the four evaluated glazing systems on indoor thermal stability over a 24-h cycle is illustrated in Fig. 8. The results indicate a consistent thermal trend across all configurations, with indoor temperatures reaching their peak between 14:00 and 16:00 and receding to a one-day minimum between 05:00 and 06:00, where temperatures converge near 24 °C.

The magnitude of the temperature peaks, however, varies significantly depending on the glazing properties:

- Single Glazed Clear Glass: Recorded the highest peak at 58 °C, demonstrating poor resistance to solar heat gain and high thermal transmittance.
- Double Glazed Clear Glass: Reduced the peak to 48 °C, illustrating the benefit of the insulating air gap in reducing conductive heat transfer.
- Low-E Coating (Vistalite sky-blue): Further mitigated the peak to 45 °C, leveraging low-emissivity properties to reflect long-wave infrared radiation.
- Solar Control Glazing (Stopray Smart 30): Achieved the most superior performance with a peak of 41 °C.

The superior performance of the Stopray Smart 30 system can be attributed to its optimized Solar Heat Gain Coefficient (SHGC), which effectively filters short-wave solar radiation before it enters the indoor space, thereby preventing the greenhouse effect observed more prominently in the clear glazing samples.

### Annual thermal dynamics and seasonal stability

**Fig. 9:**
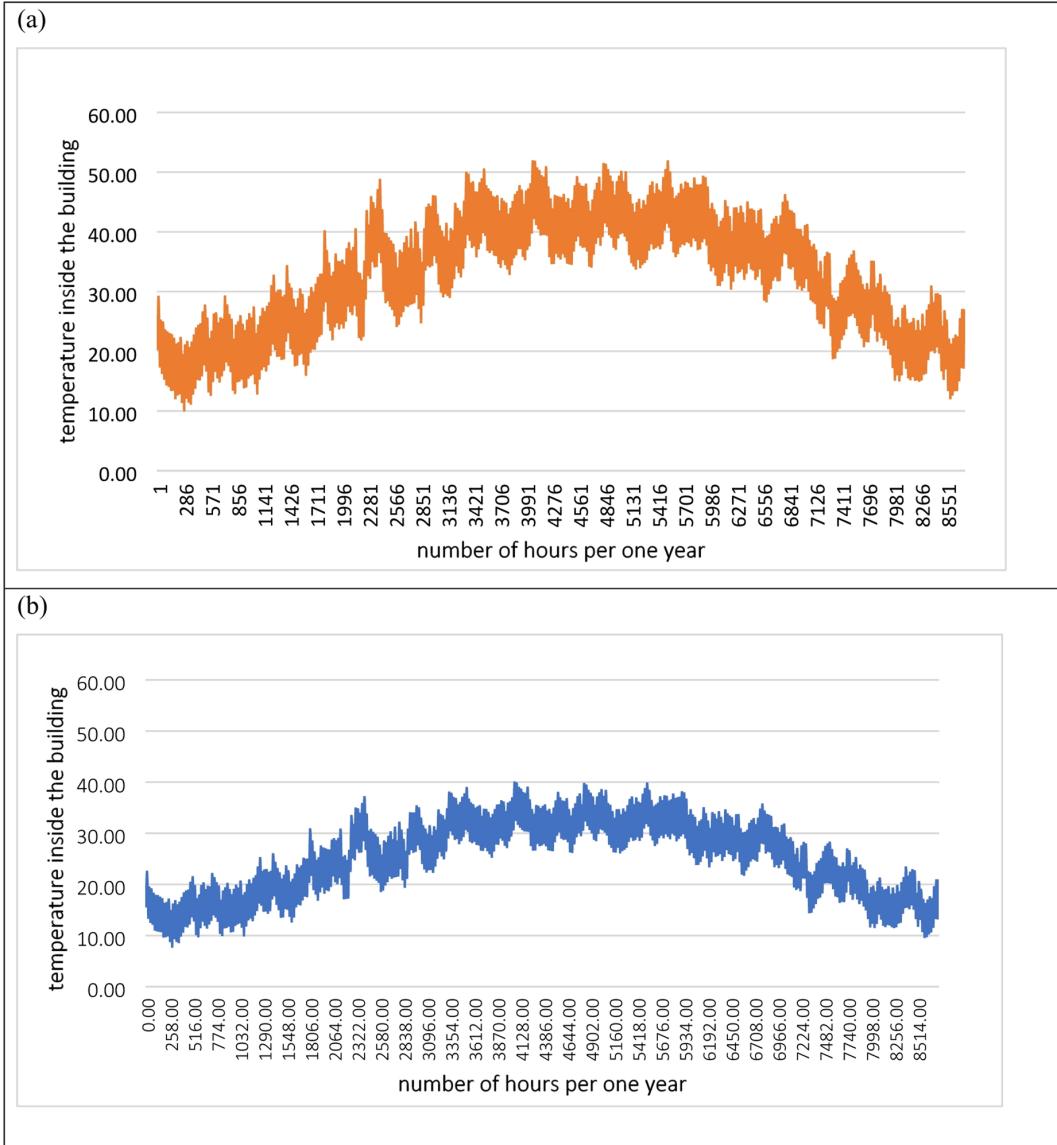
Temperature variation inside the building all over one year for (**a**) Double glazed clear glass, and (**b**) solar control glazing (Stopray Smart 30).

To assess the long-term viability of the proposed solutions, Fig. 8 presents the annual temperature variation for the double-glazed clear glass (Fig. 9a) versus the solar control glazing (Fig. 9b). The simulation model, developed in TRNSYS 17, reveals that the double-glazed clear system frequently subjects the building interior to extreme temperatures, with summer peaks reaching 45 °C.

In contrast, the Stopray Smart 30 solar control glazing maintains a much narrower temperature band throughout the year, with maximum summer temperatures restricted to 33 °C. This represents a significant enhancement in building thermal performance, approximately 22%, when compared to standard double glazing. This reduction is critical for reducing “thermal stress” on building occupants and minimizing the hours during which active cooling is required to maintain a comfortable environment.

### Cooling load analysis and energy consumption

**Fig. 10:**
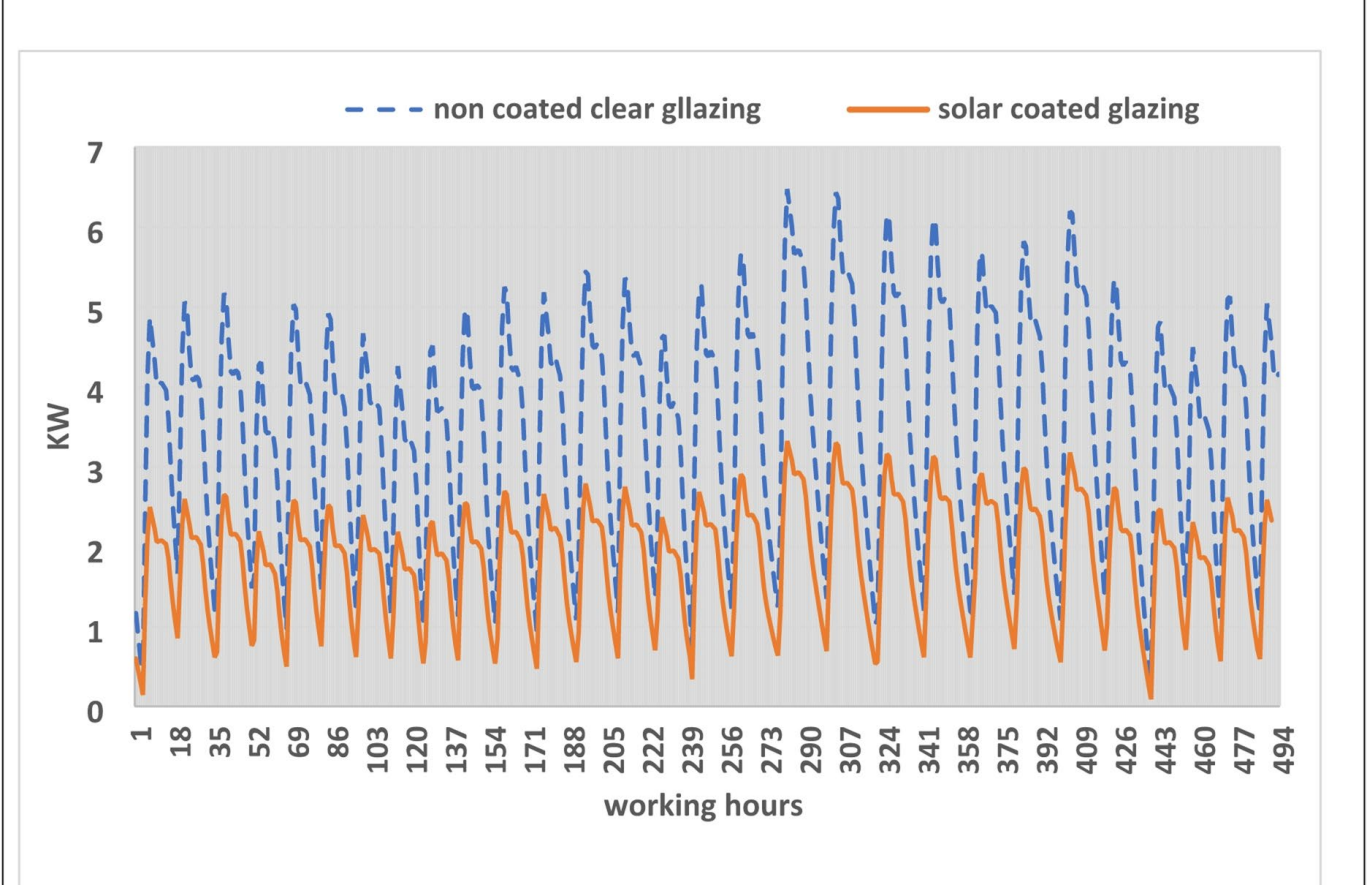
The cooling load generated on using different glazing types [author, 2025].

The practical implications of these thermal improvements are quantified in Fig. 10, which illustrates the hourly cooling load (kW) required to maintain a set-point temperature of 24 °C during the peak summer months of July and August.

- *Peak load reduction* Replacing non-coated clear glazing with solar control glazing reduces the peak cooling demand from a range of 2.5–6.5 kW down to significantly lower levels.
- *Energy consumption* The total electrical consumption for the two-month peak period was estimated at 2650 kWh for the building with non-coated clear glazing. This was reduced to 1200 kWh when using the Stopray Smart 30 system.

These results demonstrate that implementing advanced solar control glazing can reduce the cooling energy demand by more than 50% during the most demanding months. This aligns with industrial energy management goals to decarbonize the building sector and optimize HVAC system performance.

### Comparative analysis and parametric interplay

The 22% improvement in thermal performance and the 54.7% reduction in cooling energy consumption observed in this study align closely with contemporary research in hot-arid climates. For instance, similar studies conducted in the MENA region have reported energy savings ranging from 15 to 40% when transitioning from standard double glazing to high-performance solar control systems. The higher savings achieved here (54.7%) can be attributed to the extreme baseline temperature (58°C) of the non-coated clear glazing, which highlights the critical necessity of advanced coatings in regions with high solar irradiance.

The superior performance of the Stopray Smart 30 over the Vistalite sky-blue is fundamentally driven by the interaction between the Solar Heat Gain Coefficient (SHGC) and the U-value. While the U-value primarily governs conductive heat transfer, crucial for maintaining indoor-to-outdoor temperature gradients, the SHGC is the dominant factor in determining dynamic cooling loads in hot climates. In this study, the solar control glazing effectively filters the near-infrared portion of the solar spectrum, which carries approximately 50% of solar energy, before it can be absorbed by internal surfaces and re-radiated as long-wave heat. This prevents the rapid rise in sensible cooling load during peak hours (12:00–16:00), explaining the significant reduction in peak kW demand shown in Fig. 9.

### Comfort–Energy optimization and trade-offs

A critical consideration in the selection of high-performance glazing is the inherent trade-off between solar control and Visible Light Transmittance (VLT). While the Stopray Smart 30 provides the most significant cooling load reductions, its lower VLT compared to Vistalite sky-blue and clear glass may impact daylight availability.

1. *Visual comfort* The reduced VLT of solar control glazing can be beneficial in arid regions by mitigating disability glare caused by intense direct sunlight. However, it may increase the building’s dependency on artificial lighting during overcast periods or late afternoons.
2. *The luminous efficacy of glazing* The selection of Stopray Smart 30 represents a prioritized energy-first optimization strategy. In industrial or office settings where cooling dominates the energy budget, the energy saved by reducing HVAC loads typically far outweighs the minor increase in electrical lighting demand.
3. *Dynamic loads* Unlike the U-value, which remains relatively static, the cooling load is dynamic and follows the solar path. The solar control glazing acts as a passive peak-shaver, reducing the volatility of the indoor thermal environment and allowing for more stable HVAC operation, which enhances the longevity of the cooling equipment.

By positioning the results within this comfort–energy discourse, it becomes evident that while Vistalite sky-blue offers a balanced approach for residential applications where daylighting is prioritized, Stopray Smart 30 is the optimal choice for large-scale energy intensive buildings seeking to minimize peak electrical demand and carbon intensity.

### Environmental impact and carbon footprint analysis

The significant reductions in cooling energy demand achieved by the high-performance glazing systems directly contribute to the mitigation of the building’s environmental impact. To quantify this effect, the electrical energy savings during the peak summer months (July and August) were converted into carbon dioxide (CO_2_) emissions. This calculation utilized a standard grid emission factor for the local context of approximately 0.50 kg CO_2_/kWh. This analysis is essential for evaluating the building’s alignment with national decarbonization strategies and the transition toward net-zero energy operations.

As shown in Table 2, the baseline configuration using non-coated clear glazing resulted in an estimated 1325 kg of CO_2_ emissions during the two-month peak period. In contrast, the implementation of Stopray Smart 30 solar control glazing reduced these emissions to 600 kg, representing a substantial carbon footprint reduction of 54.7%. The Vistalite sky-blue (Low-E) system also demonstrated a robust environmental benefit, achieving a 41.5% reduction in emissions compared to the baseline.

**Table 2:** Comparative analysis of energy consumption and carbon footprint (July–ugust).

Glazing type	Energy consumption (kWh)	CO2 emissions (kg)	CO2 savings (kg)	Carbon reduction (%)
Non-coated clear (Baseline)	2650	1325	–	–
Vistalite sky-blue (Low-E)	1550*	775	550	41.5%
Stopray smart 30 (solar control)	1200	600	725	54.7%

*Estimated based on the proportional reduction in peak indoor temperature (45°C) relative to the baseline (58°C) and solar control (41 °C) performance.

The results indicate that selecting advanced glazing technologies is a critical lever for reducing the “carbon intensity” of the built environment. Beyond the direct reduction in operational emissions, the lowered peak cooling loads (kW) illustrated in Fig. 9 suggest that HVAC systems can be appropriately downsized. This downsizing potentially leads to further “embodied carbon” savings during the construction and procurement phases, as well as reduced indirect emissions from refrigerant leakage over the system’s lifecycle. Consequently, the integration of solar control glazing stands as a highly effective passive strategy for industrial energy management and large-scale decarbonization of the building sector.

## Conclusion

In the current study, the thermal efficiency of different glazing units in hot arid region (Cairo, Egypt) was investigated experimentally. A CFD study was also carried out using TRNSYS 17 software to validate the experimental results and to evaluate the impact of different glazing units on the thermal efficiency of an office building.

The experimental investigations showed that, the temperature of the constructed concrete chamber was decreased by 17^O^C when using solar control glazing (Stopray Smart 30) instead of normal clear glass. The thermal efficiency for concrete chamber was enhanced by 17, 22, and 29% for double glazed glass, low E coating glazing (Vistalite sky-blue), and solar control glazing (Stopray Smart 30), respectively.

Simulation results are in good agreement for practical application where the difference between experimental and simulation results did not exceed 9%. A significant enhancement (by 22%) in the building thermal efficiency was noticed when using solar control glazing (Stopray Smart 30). Besides, the cooling energy consumption was reduced by 50% compared to buildings with regular glazing windows.

Finally, optimizing the use of smart glass contributes to more sustainable and energy-efficient buildings that directly affect energy consumption, operational costs, environmental footprints and CO2 emissions.

## Data Availability

The data that support the findings of this study are available upon request from the corresponding author.
